# Next Generation Sequencing of Actinobacteria for the Discovery of Novel Natural Products

**DOI:** 10.3390/md14040078

**Published:** 2016-04-13

**Authors:** Juan Pablo Gomez-Escribano, Silke Alt, Mervyn J. Bibb

**Affiliations:** Department of Molecular Microbiology, John Innes Centre, Norwich Research Park, Norwich, NR4 7UH, UK; silke.alt@jic.ac.uk (S.A.); mervyn.bibb@jic.ac.uk (M.J.B.)

**Keywords:** actinomycetes, *Streptomyces*, genome mining, PacBio, Illumina, next generation sequencing

## Abstract

Like many fields of the biosciences, actinomycete natural products research has been revolutionised by next-generation DNA sequencing (NGS). Hundreds of new genome sequences from actinobacteria are made public every year, many of them as a result of projects aimed at identifying new natural products and their biosynthetic pathways through genome mining. Advances in these technologies in the last five years have meant not only a reduction in the cost of whole genome sequencing, but also a substantial increase in the quality of the data, having moved from obtaining a draft genome sequence comprised of several hundred short contigs, sometimes of doubtful reliability, to the possibility of obtaining an almost complete and accurate chromosome sequence in a single contig, allowing a detailed study of gene clusters and the design of strategies for refactoring and full gene cluster synthesis. The impact that these technologies are having in the discovery and study of natural products from actinobacteria, including those from the marine environment, is only starting to be realised. In this review we provide a historical perspective of the field, analyse the strengths and limitations of the most relevant technologies, and share the insights acquired during our genome mining projects.

## 1. Introduction

Actinobacteria produce more than 70% of all natural product scaffolds used for the manufacture of clinically-relevant anti-infectives [1]. During the final decades of the last century, efforts to discover new anti-infectives of microbial origin were almost abandoned; however, the beginning of this century has seen a return to the search for novel bioactive natural products from actinobacteria, due to advances in cultivation and activity screening [2,3] and, in particular for this review, the realisation of the biosynthetic potential of actinobacteria, encoded in the genome but not expressed as compounds, and the development of genetic approaches to access this hidden potential [3,4].

During the sequencing of the *Streptomyces coelicolor* genome in the late 1990s [5] it became evident that actinomycetes carry the genetic potential to produce many more natural products than those detected under laboratory conditions, and during the following years many previously-unknown metabolites produced by *S. coelicolor* were identified and characterised [6]. It was realised then that access to the genome sequence of a strain could be used to unlock the biosynthetic potential of the micro-organism using different molecular genetic approaches [1,6,7,8] in a strategy that has been called “genome mining” [7]. However, in the early 2000s the only automated technique for DNA sequencing was the dideoxynucleotide method developed by Sanger and co-workers [9]; even with the developments reached in the 2000s, including those in computing and assembly algorithms [10], Sanger sequencing was too expensive and labour intensive to provide sufficient coverage for a routine whole-genome shotgun approach, requiring the creation, sorting, and sequencing of genomic libraries prior to full genome assembly [5,10].

Since the late 2000s we have seen the continuous release of new DNA sequencing technologies that have pushed forward both sequencing capacity and accuracy, and lowered the cost per sequenced nucleotide. These technologies are referred to as next-generation sequencing (NGS) and are defined as “non-Sanger-based, high-throughput, and eliminating the need for fragment-cloning and amplification in *Escherichia coli* prior to sequencing” (adapted from [11]). NGS technologies make affordable the high-throughput sequencing of bacterial genomes which, when coupled with a continuous advance in computing algorithms and databases for the automated scanning and annotation of specialised metabolite gene clusters like AntiSMASH [12] and MIBiG [13], are enabling the continuous discovery and study of natural products biosynthetic pathways by genome mining.

The relatively recent realisation that actinobacteria from our oceans and seas are much more abundant than previously thought provides a new opportunity for the discovery of drugs from the marine environment. Coupled with the difficulty of growing some of these organisms at a large scale, the sequence-based approach to natural product discovery described here should be particularly pertinent and useful.

## 2. A Short Walk through NGS Technologies

The first NGS technologies to appear, referred to as second-generation sequencing (SGS), relied on cycles of the termination of DNA polymerisation and recording of the incorporated nucleotides in each cycle. The first SGS technology to be commercialised, in mid-2005, was 454 pyrosequencing (by 454 Life Sciences, now a subsidiary of Roche Diagnostics) [14], followed in 2006 by the reversible-terminator chemistry of Solexa/Illumina (by Solexa Ltd., now Illumina Inc.) [15] which, because of its lower cost, high throughput, and accuracy [16], has become the first choice sequencing technology across many fields of research and medical diagnostics. Other SGS technologies have been commercialised but have not attained the popularity of these two. Examples that have been used for actinobacteria genome sequencing are SOLiD (released in 2006 by Applied Biosystems Inc., now Thermo Fisher Scientific/Life Technologies) and Ion Torrent (released in 2010 by Ion Torrent Systems Inc., now Thermo Fisher Scientific/Life Technologies).

The year 2011 saw the commercialisation of the first third-generation sequencing (TGS) technology: Single Molecule Real Time (SMRT) (by Pacific Biosciences of California, Inc.) usually referred to as “PacBio” [17]. Another TGS technology currently in development, but already very promising, is Nanopore DNA sequencing (by Oxford Nanopore Technologies Ltd.); this platform is accessible through an early access program [18]; early reports indicate that the technology, despite read accuracy under 70%, is already useful for scaffolding, thanks to read lengths of over 1 kb and up to 98 kb; however, the application to actinobacterial genome sequencing is still heavily hampered by the high mol% G+C of the organisms [19,20].

TGS technologies, as opposed to SGS, rely on sequencing single molecules without amplification (which can create problems with even genome representation) and in real-time (no cycles of polymerisation/termination) and are capable of providing read lengths of several kilobases [21] facilitating the assembly of whole-genome shotgun projects.

## 3. Challenges of Actinobacterial Genomics

The high mol% G+C content of actinomycete genomes poses difficulties not just for the sequencing technology itself but also to the computing algorithms used for the assembly [22], although many of the errors and sequencing biases due to mol% G+C bias have been lessened by improvements in library preparation [23,24]. A more specific problem is presented by the linear chromosome and plasmids of many important actinobacteria, like the streptomycetes, with long terminal inverted repeats that can reach over one megabase [25], impossible to resolve with current sequencing technologies. In addition, extraction of high molecular weight DNA of the high quality required for NGS library construction, and in particular of TGS, is not trivial and in many cases currently not feasible, especially from actinobacteria difficult to culture and resilient to cell wall digestion.

**Figure 1:**
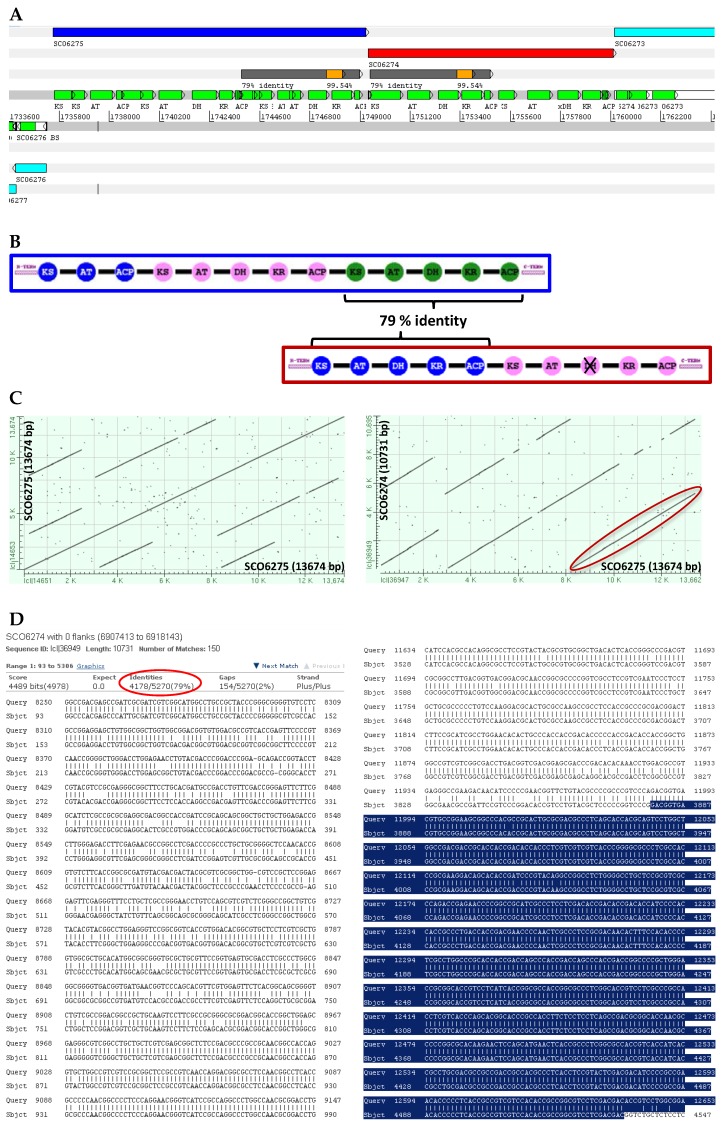
Example to illustrate the problem of intergenic and intragenic sequence identity in genes encoding modular polyketide synthases. (**A**) Screenshot from Artemis software [31] showing the genetic organisation of the first two PKS genes of the coelimycin gene cluster; the highlighted segments correspond to the last module of Sco6275 and first of Sco6274 (**B**) which share a sequence identity of 79% over 5 kb (grey) and more than 99.5% identity over 650 bp (orange). A closer look at the nucleotide sequence of these genes by BLAST (dot plots and alignments generated at NCBI server) shows the intragenic (**C**, left) and intergenic (**C**, right) regions of high identity, and the nucleotide alignment of the region in grey (**D**, left) and orange (**D**, right, highlighted in blue). KS, ketosynthase; AT, acyltransferase; DH, dehydratase; KR, ketoreductase; ACP, acyl-carrier protein.

Many of the most relevant natural products belong to the chemical families of Type I polyketides and non-ribosomal peptides [26]. The backbone of these compounds is synthesised by large enzymes, polyketide synthases (PKS), and non-ribosomal peptide synthetases (NRPS), which consist of a highly-conserved modular enzymatic architecture that is reflected at the nucleotide sequence level by highly similar intragenic and intergenic tandem repeats, frequently spanning over 700 bp; e.g., *S. coelicolor* coelimycin PKS gene *sco6274* positions 3879–4533 and *sco6275* 11986–12639 share 99% intergenic identity (Figure 1); and the calcium-dependent antibiotic NRPS gene *sco3230*, positions 13187–14121 and 16307–17241, share 95% intragenic identity. These repeats are, in many cases, longer than the read-length of all SGS technologies, making it very difficult, if not impossible, to correctly assemble these very important gene clusters.

## 4. The Read-Length Problem

When we talk about “assembly” we mean the determination of the correct order of the reads, the thousands of short overlapping fragments in which the whole genome sequence would be represented (see [27] for a good review). Due to simple statistics, the longer and more accurate the reads are, the longer and more specific the overlap between two reads will be and, therefore, a more reliable assembly can be computed [28].

Of the SGS technologies, 454 has provided, consistently, the longest read length and dominated actinobacterial genome sequencing until 2012 when Illumina took over with an increasing high-accuracy read-length, together with the highest output and lowest cost per base. Currently, with maximum paired-end (*i.e*., both ends of the same DNA molecule are sequenced) read lengths of around 2 × 150 nt for Illumina’s highest throughput HiSeq sequencers, and around 2 × 300 nt for the lower throughput MiSeq (source: www.Illumina.com; accessed on October 2015), Illumina technology can typically deliver a maximum of 500 nt of contiguous reliable sequence, insufficient to resolve the highly repetitive PKS and NRPS genes, ribosomal RNA operons (which span about 5.5 kb), and terminal inverted repeats. Of the other SGS technologies, only 454-pyrosequencing provides longer read lengths, 700 nt and up to 1 kb according to the manufacturer (GS FLX+ instrument; source: http://454.com; accessed on October 2015). Ion Torrent is currently offering up to 400 nt reads and presents an alternative to Illumina for *de novo* sequencing of small genomes [29].

This situation has changed dramatically with the advent of TGS technologies. PacBio SMRT, the only TGS technology currently commercialised, is still unique in its ability to resolve highly-similar repetitive sequences thanks to an average read-length of over 10 kb [30] at an affordable 100 fold coverage required, not just for a reliable assembly, but also for an accurate base-calling of the consensus sequence.

## 5. Historical Perspective of Actinobacterial Genome Sequencing

The first actinobacterial genome fully sequenced was that of *Mycobacterium tuberculosis* [32], and the first genome of an actinobacterium relevant for natural products was that of the model streptomycete *S. coelicolor* [5], followed by the industrially important *Streptomyces avermitilis* [33], both using Sanger sequencing. As standardly practiced at the time, the *S. coelicolor* genome was assembled by sequencing an ordered cosmid and BAC genomic library. In contrast, sequencing of the genome of *S. avermitilis* was first attempted using a whole-genome shotgun approach; the published article provides enough detail for us to grasp the difficulties of a whole-genome shotgun approach only a decade ago; two genome contigs of 9,025,608 nt and 94,287 nt (the chromosome and a linear plasmid, respectively; a total of 9,119,895 nt) were assembled initially from 186,619 sequences from random ~2kb fragments (estimating 600 nt of high-quality base-calls, (186,619 × 600) nt/9,119,895 nt = 12.3 fold coverage); this data was not sufficient for a good quality assembly and the authors had to rely on the end-sequencing of more than 10,000 cosmids from a genomic library, over a hundred full cosmid inserts, and 162 PCR-product sequences, altogether reaching 13.3 fold coverage with directed gap filling [33]. This example demonstrates that shotgun sequencing with exclusive use of Sanger-based techniques was extremely laborious and not sufficient for efficient full genome shotgun assembly.

The SGS technologies did allow shotgun sequencing of whole genomes at much higher coverage, but the short read-length combined with the intrinsic difficulties of actinobacterial genomes hampered the assembly of full replicons in single contigs. Due to the longer read-length, 454-pyrosequencing has been the technology of choice for achieving a more complete genome assembly, but full replicon assembly into a single contig has required the use of Sanger end-sequencing of large-insert genomic libraries and directed gap-filling by PCR-amplification. Examples are the sequencing of the genomes of *Streptomyces hygroscopicus* 5008, in which Illumina data was used to correct base-call errors [34], and of *Streptomyces albus* J1074 for which, even with a 377 fold coverage from combined Illumina-454 sequencing data of three libraries of different insert-size, a large BAC library was end-sequenced and chromosome-walking used to fill gaps and merge the initial 76 contigs [35].

## 6. The Explosion of Genome Mining

**Figure 2:**
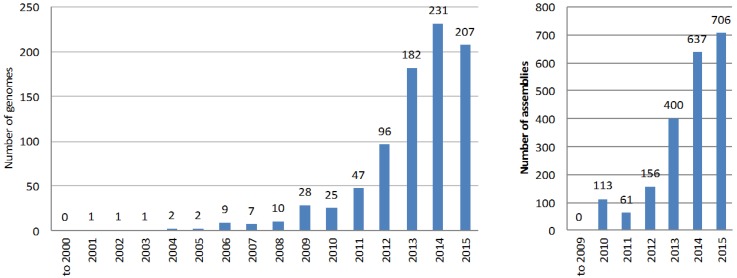
Number of genomes (left) and assemblies (right) of actinobacteria species relevant to natural products research deposited at NCBI databases per year.

Despite the difficulty in completing an accurate full genome sequence of an actinobacterium, the number of available genome assemblies (of different completeness and quality level) in the databases has grown exponentially during the past decade (Figure 2). The main reason is that a complete and accurate genome sequence is not a prerequisite for the application of the genome mining approach to natural product discovery; multi-contig genome drafts can be equally useful when it comes to identifying the gene cluster that drives the synthesis of a known compound, or gene clusters for the biosynthesis of unknown but potentially interesting natural products. The most common automated pipeline for searching and annotating natural products biosynthetic gene clusters, AntiSMASH [12], can easily deal with multi-contig genome assemblies.

Even in the early days of Solexa technology, the information obtained could suffice for designing strategies for genome mining. For the identification and cloning of the microbisporicin biosynthetic gene cluster, almost 900 Mb of sequence data for the *Microbispora corallina* genome was obtained, but despite the high coverage (greater than 100×) the short read length at the time, only 36 nt, allowed only a very fragmented assembly (see Table 1). However, this provided enough information to identify putative homologs of lantibiotic biosynthetic genes, to design probes for Southern Blotting, and to successfully screen a cosmid library to obtain the entire gene cluster [36,37]. Genome sequencing with Solexa was also crucial in the identification and cloning of the cypemycin gene cluster after strategies based on hybridisation screening of a cosmid library were unsuccessful [38].

At the same time, 454 pyrosequencing, with its longer reads (200–400 nt at the time), provided a clear advantage for actinobacterial genome assembly; sequencing of *M. corallina* genome with one quarter of a run of 454 yielded much less data than Solexa, but led to an assembly with fewer and longer contigs that allowed the identification of more putative lantibiotic biosynthetic genes and, crucially, the gene encoding the putative precursor peptide by using the amino acid sequence as a query for a tBLASTn search [37]. The planosporicin [39,40], tunicamycin [41], and bottromycin [42] biosynthetic gene clusters were also cloned on the basis of draft genome sequences obtained with 454 (see Table 1 for a summary of our lab’s experience).

Partial genome sequence information has also been used to design successful strategies to obtain or identify the unknown metabolic product of a biosynthetic gene cluster. Three main strategies are pursued: the activation of the expression of the gene cluster in the natural producer, cloning and expression in a heterologous host, and mutation followed by metabolite profiling. An elegant example of the first approach is the identification and characterisation of stambomycin; Laureti and co-workers found a cryptic gene cluster (*i.e*., without a known product) encoding the biosynthesis of an unknown polyketide in the draft genome sequence of *Streptomyces ambofaciens* and, after inferring the transcriptional regulatory network, they induced the production of the metabolic product by constitutive expression of a cluster-situated gene encoding a transcriptional activator of the LAL family [44]. A similar example is the discovery and characterisation of ansamycin compounds with novel chemistry from *Streptomyces* sp. LZ35 [45]. An example of the second approach is the characterisation of grisemycin, a linaridin from *Streptomyces griseus* IFO 13,350, which was purified after heterologous expression of the cloned gene cluster [46]. The third approach can be used when the gene cluster is expressed under laboratory culture conditions, but its product is not known: the genome sequence can be used to design gene knock-outs that, together with comparative metabolite profiling of the producing and non-producing strains, allows the identification of the metabolic product (e.g., [47]).

Thus, despite the large number of contigs, and most likely many misassembly issues, draft genome sequences can, indeed, be sufficient to successfully identify new natural products or the biosynthetic gene clusters of known compounds. Dozens of research groups across the globe have adopted one or more of these approaches, with more than 120 papers on this topic published by the end of 2015 (number of records found in PubMed with the search string “(“genome mining”) AND (streptomyces OR actinobacteria OR actinomycetes OR streptomycetes)”) of which 70 were in the last two years (note that since PubMed does not index many journals relevant to the field, this is likely to be an under estimate of the number of relevant publications). Therefore, it should not be surprising that the nucleotide sequence databases contain over 2000 genome assemblies of actinobacterial species.

**Figure 3:**
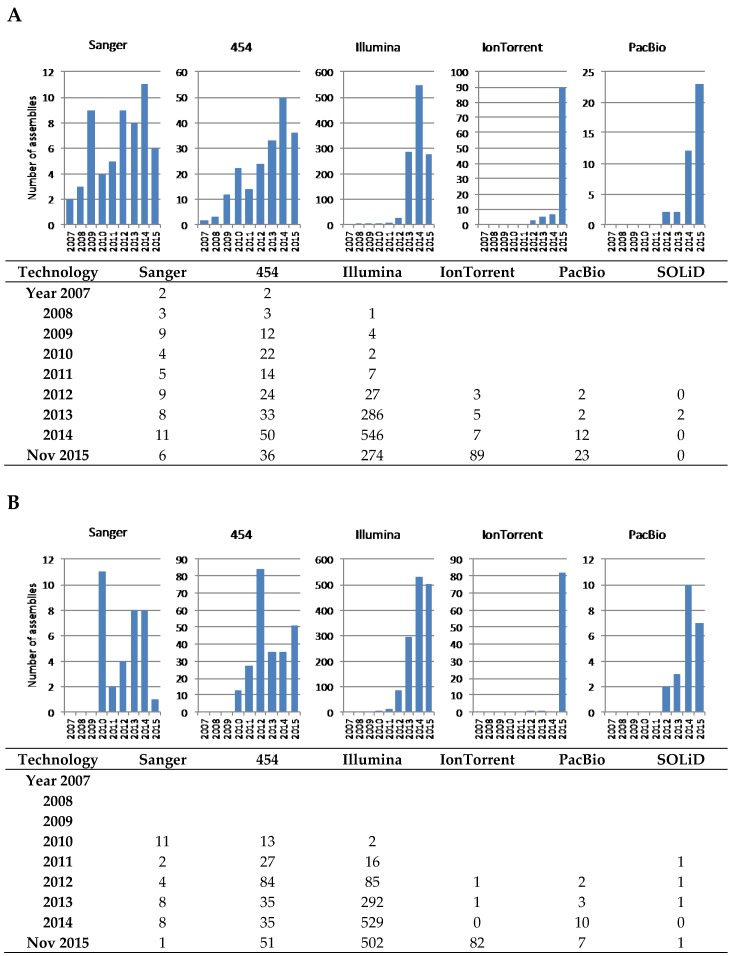
Distribution of assemblies included in the PATRIC (**A**) and NCBI (**B**) databases at the end of 2015, according to the technology used. Note that some assemblies have been obtained with a combination of several technologies and have been accounted for in each of them. Prior to 2007 there are 18 assemblies not included in the figure because the sequencing technology is not stated, although most certainly all were obtained by Sanger sequencing, or at least the 15 with complete status.

Although the situation is improving with more stringent and precise requirements during sequence submission, it is still difficult to obtain accurate characteristics about genome sequences available in public databases. There is also confusion about the terminology used by each database; e.g., NCBI hosts a database named “Assembly” that contains released sequences at different stages of completion; entries in the “Genome” database contain “assemblies” grouped at the species level, even with different strains in the same “genome” entry. It is also difficult to easily search for completed genomes, rather than drafts with hundreds of contigs; even metagenomic projects are included as a single entry in both “Genome” and “Assembly” databases. Even more difficult is to search by sequencing technology, due sometimes to the lack of information or to the use of different terms by different researchers when submitting to databases. Thus, while we have tried to ensure the accuracy of the numbers presented in the following paragraphs and Figure 2 and Figure 3, they are not intended to be an absolutely precise description of the databases content.

At the end of 2015, a search for “actinobacteria” in the NCBI databases (search string “txid1760 [Organism:exp]”) identified 1065 genomes and 7057 assemblies. Of these, 83 genomes and 4268 assemblies (over 60%) belong to *Mycobacterium* species alone, mostly to clinical isolates of *M. tuberculosis* (with 3635 assemblies). Since this review focuses on actinobacteria relevant for natural products research, genomes and assemblies from species belonging to the clinically-important genera *Mycobacterium* (Taxonomy ID: 1763), *Propionibacterium* (Taxonomy ID: 1743), *Gardnerella* (Taxonomy ID: 2701), and *Corynebacterium* (Taxonomy ID: 1716), and species of *Bifidobacterium* (Taxonomy ID: 1678), a genus that is becoming more abundant in databases because of the projects on human microflora, were filtered-out from the searches; this is not because of lack of potential relevance to natural products research but because the large number of assemblies would introduce a misleading bias in the analysis reported here. The result was that there are 849 genomes and 2073 assemblies of actinobacteria strains potentially relevant to natural products research available in the NCBI databases (Figure 2). Of the 2073 assemblies, 1865 assemblies are only completed to contig or scaffold level.

The PATRIC database [48] search tool allows a more comprehensive and precise search of bacterial genomes and, importantly, includes the completion achieved and sequencing technology used. At the time of writing, the newest entries in the PATRIC database were dated November 2015, and contained 7148 genome assemblies of actinobacteria (taxon ID 1760). Almost half corresponded to clinical isolates of *Mycobacterium tuberculosis* (3785) and a total of 5330 corresponded to the clinically-relevant strains of *Mycobacterium*, *Corynebacterium*, and the increasingly represented *Gardnerella*, *Propionibacterium*, and *Bifidobacterium* genera. As explained above, in order to provide a more faithful representation of the genome sequencing projects relevant to natural products research, all assemblies of strains belonging to these genera were discarded from the statistics shown in this review; while many of these actinobacteria might be relevant to natural products research, the low number of actual species filtered out is not likely to influence the conclusions of this review.

PATRIC allows a more comprehensive perspective on the use of technologies than NCBI (which seems not to contain searchable information prior to 2010); however, it is still not easy to analyse the information (the names used vary, e.g., 454 can be referred to with just “GS FLX”) and make any search and sorting very difficult, and there were 118 genomes sequenced with more than one technology, almost always involving 454 and Illumina, and there were over 300 entries without an associated technology) and, consequently, we chose to download the Actinobacteria genomes table and analyse it manually. The picture painted by our analysis (Figure 3) shows that Illumina is by far the most commonly used technology, due to the low cost per nucleotide, the very high throughput, and the wide availability of suppliers. As mentioned above, the longer reads of 454 pyrosequencing made this technology, despite its higher cost, the choice when a more precise assembly was desired, in particular for PKS and NRPS gene clusters, and so it was the main technology used until just two years ago, when Illumina took off. A continuous presence of Sanger sequencing reflects the use of this technology to complement the limitations of NGS when the goal is to obtain a finished genome; end-sequencing of genomic library clones and sequencing of PCR amplicons to fill gaps in the assembly are the most common applications of Sanger. SOLiD technology has been a very minor player with only 2–4 entries in PATRIC and NCBI, respectively. Interestingly, Ion Torrent seems to have gained momentum in the past year; since most of the submitted projects originated at the same institution, this may not reflect wider adoption, although improvements in library construction with high mol% G+C DNA [49] and bioinformatics [50] might help this technology to be more widely embraced in the future.

Assemblies obtained using TGS PacBio were submitted to the databases from 2012, but the first examples made use primarily of Illumina and 454 SGS, using limited coverage obtained with PacBio for scaffolding of contigs and gap closing [51,52].

## 7. Pacific Biosciences SMRT Platform

The first streptomycete genome that appears in the literature as sequenced with PacBio is an unfinished sequence of *Streptomyces* sp. strain Mg1 [53]; despite the relatively low coverage (20×) most of the chromosome was obtained as a single contig (accession GCA_000412265.1); however, the authors did not present a detailed analysis and only highlighted the improvement of the single contig as opposed to a previous 466-contigs assembly released by the Broad Institute (ABJF00000000) using Illumina; a more detailed comparison of both assemblies was included in a review article by Harrison and Studholme [54] but, since Illumina technology had evolved enormously during the five years that separate both assemblies, the comparison may be misleading.

Our group has recently published the sequencing of the genome of *Streptomyces* *leeuwenhoekii* C34, a strain isolated from the Atacama Desert in Chile [43]. The genome was sequenced and assembled independently with PacBio and Illumina MiSeq (paired-end 250 nt reads) between August and November 2013. In addition, PAC clones from a genomic library were also sequenced with 454 Junior [55], and we also have sequencing information obtained with Sanger during the study of specific gene clusters ([55] and Razmilic, manuscript in preparation). These data, obtained at almost the same time, provide a useful comparison of the relative strengths and limitations of all three technologies; some of these were also discussed in [43] but a more detailed view will be provided here.

## 8. PKS Modularity can be Resolved with PacBio

The genome of *S. leeuwenhoekii* C34 was first assembled by Busarakam and co-workers using Illumina 100 nt paired-end reads in to 658 contigs totalling 7.86 Mb [56]; analysis of this draft revealed numerous miss-assembly issues in the PKS genes, not surprising bearing in mind the short read length used [43,55]. We also sequenced this genome with PacBio RSII. After a quality-filter of the reads, we obtained almost 1 Gb of sequence data from 2 SMRT cells (plus a first cell that failed and generated only 77 Mb of sequence, so the full assembly was essentially derived from just 2 SMRT cells) which was assembled into just 3 contigs of 7.9 Mb, 95 kb and 10 kb. Oddly, the smallest contig matched a stretch of the largest contig with over 90% identity; these small and error-prone contigs have been observed in other PacBio sequencing projects (e.g., [57]). The largest contig contained an almost complete chromosome, in a single uninterrupted sequence without the “Ns” typical of scaffolding with Illumina sequencing; only the ends of the terminal inverted repeats were not fully sequenced.

Almost concomitantly, we sequenced this genome with Illumina MiSeq 250 nt paired-end reads, obtained 1.25 Gb of data of which 712 Mb were assembled into 279 contigs, merged into 175 scaffolds, totalling 8.1 Mb. This assembly had a striking similarity with the PacBio assembly, and most of the studied PKS genes fitted the expected modularity ([55] and unpublished data). However, even with such high coverage, some PKS modules had been misassembled [43].

## 9. Identification of Circular or Linear Replicons

The assemblers produce linear molecules, but do not make decisions on the topology of the DNA molecule. Due to the long reads of PacBio, some reads will run into the terminal inverted repeats (TIRs) of linear replicons, allowing the identification of at least the beginning of the TIRs [43]; similarly, the presence of direct repeats at the ends of the contig will undoubtedly indicate that this is a circular replicon and allow the circularisation of the molecule to remove the duplicated non-existent sequence [43]. Illumina did an excellent job at assembling the circular plasmid into a single molecule, but without the direct terminal repeat of the PacBio contig it would not have been possible to identify it as a circular molecule. Another interesting finding was that PacBio could read through stretches of sequence difficult or impossible to read even by Sanger sequencing, apparently due to the formation of complex secondary structures (Figure S2).

## 10. Limitations of PacBio

### 10.1. Insertions and Deletions: Shifts in the Reading Frame

**Figure 4:**
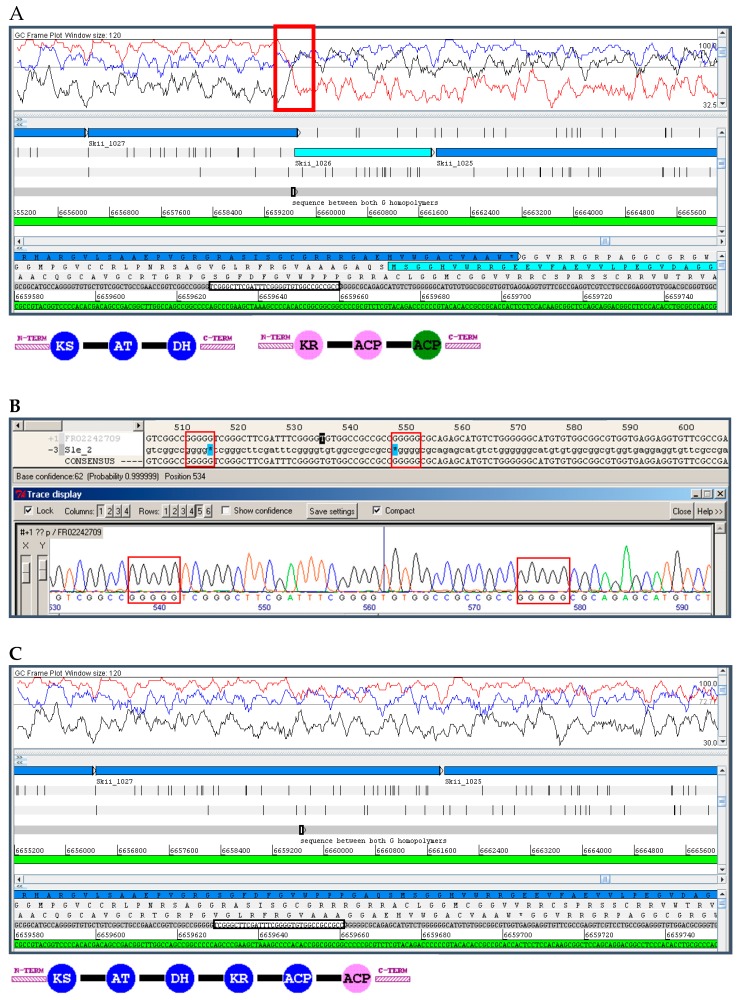

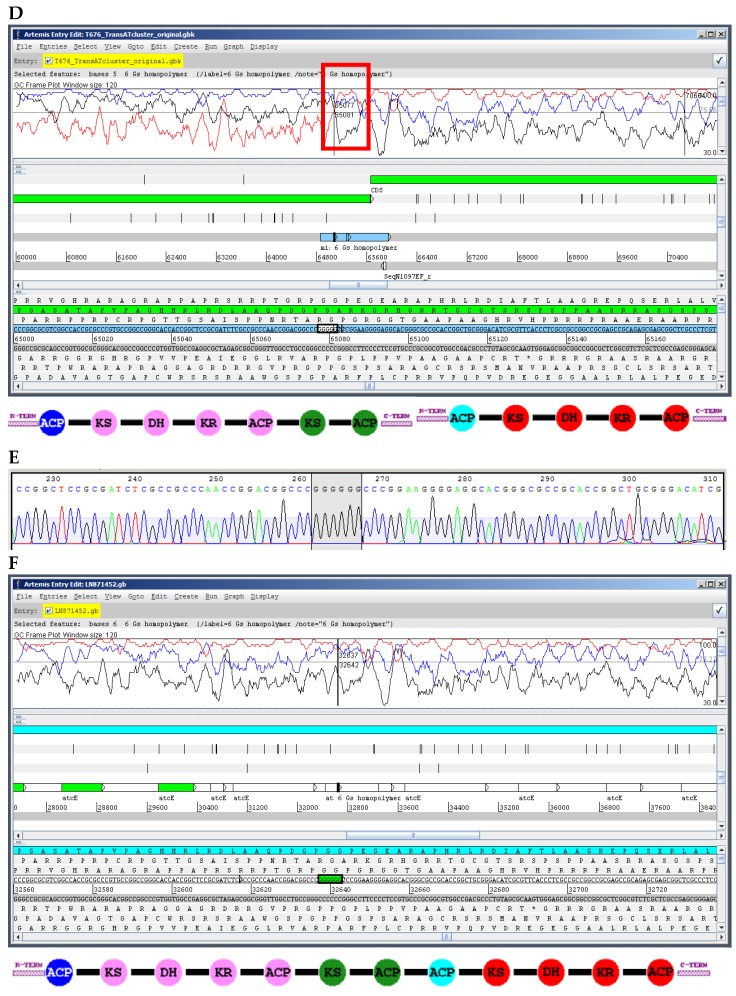
Illustration of shifts in the reading frame caused by omission of one nucleotide in homopolymers of G or C in PacBio assemblies, and their effect on the modularity of PKS genes of the chaxamycin [43,55] (**A**–**C**) and anthracimycin [57] (**D**–**F**) gene clusters. **A**,**D**. Original PacBio sequence showing the frame-shift (red box) and the break in the modularity of the PKS domains; in A, the frame shift is obvious since it splits the module of PKS domains into two protein coding sequences; in **D**, while each PKS protein exhibits an appropriate modular domain structure (non-canonical domain arrangements are not unusual in *trans*-AT PKSs [57]) note that the 3’ end of the first protein coding sequence continues in the wrong reading frame which would correspond to a highly non-canonical codon usage. **E**. Sanger sequencing of the affected region showing the affected homopolymer; in the chaxamycin gene cluster these errors had been also corrected with Illumina reads [43]; in the anthracimycin example, the entire region in the cyan box (**D**) was sequenced with Sanger, and the only error found was the one shown in **E**. **C**,**F**. Organisation of the PKS gene and domain modularity after error correction. PKS domains identified with the SBSPKS server [63]: KS, ketosynthase; AT, acyltransferase; DH, dehydratase; KR, ketoreductase; ACP, acyl-carrier protein; Note that there is a methyltransferase domain in the last module that has not been identified by SBSPKS.

The main known limitation of PacBio in actinobacteria is the resolution of G or C homopolymers; in our experience, the final consensus sequence tends to miss a G or C, perhaps reflecting an issue with the assembly algorithm rather than a limitation of the current sequencing chemistry; if so, it might be possible to fix this in future releases of the software. We observed the opposite problem with the 454 technology, where we have observed a consistent insertion of a G or C in homopolymers (Figure S1). In both cases, the insertion or deletion causes a shift in the reading frame within a protein coding sequence (PCS), which is easily identifiable by studying the “GC Frame plot”. Due to the high mol% G+C of actinobacterial DNA, there is a biased nucleotide composition at each of the three positions of a codon in a PCS: G or C are present at the third position in over 90% of codons, while they are in the second position in only around 50% of codons, and in the first position in about 70% of codons [58]. The software FRAME plots three lines corresponding to the mol% G+C of each of the three positions in the codons contained within a selected window-size [59]; a change in the distribution of the three lines (a crossing of lines) within a protein coding sequence is indicative of a shift in the reading frame caused by an insertion or deletion (Figure 4). Frame-shifts are usually easy to identify, unless they occur at the beginning or end of the PCS with a start or stop codon in proximity. If the encoded protein shows closely-related homologous proteins in the databases, a BLAST search easily identifies frame-shifts as well and can be used to confirm the FRAME plot analysis.

The insertions and deletions can be corrected by means of directed PCR amplification and Sanger sequencing (Figure 4) or at a genome-wide scale by deep-sequencing with Illumina and mapping of reads to the PacBio assembly [43,60,61]. In any case, they are easily addressable and do not pose such a problem as misassembly of PKS modules.

### 10.2. Sequence Missing from the Final Assembly

The most striking finding during comparison of our PacBio and Illumina assemblies was sequence missing from the PacBio assembly that was present in the pre-assembled data. During sequencing of the *S. leeuwenhoekii* C34 genome we found that over 130 kb of sequence, most likely representing a linear plasmid, was present in the Illumina but not the PacBio assembly; however, this sequence was present in the so called PacBio corrected or pre-assembled reads, an intermediate step in the PacBio assembly pipeline [62]; the reason why this data was not assembled is still not known, but comparison of recent assemblies obtained from the same data with version 2 or 3 of the HGAP pipeline suggests that this issue has been resolved in HGAP3 (unpublished data). Another piece of sequence missing from the PacBio assembly, and also from the raw data originated from the end of linear replicons; we found that Illumina collected about 5 kb of additional unique telomere sequence located towards the end of the chromosome than PacBio; this may simply reflect the longer insert library size (over 20 kb) used for PacBio compared with the 500 bp used for Illumina, diminishing coverage of the terminal sequences. In both cases, it is important to stress that the missing sequence at the end of the chromosome, and the almost 130 kb likely linear plasmid (containing an interesting biosynthetic gene cluster), would not have been identified had we not also assembled the Illumina data, and instead used just the reads to improve the accuracy of the PacBio assembly.

## 11. Application to Actinobacteria from Marine Environments

The marine environment has proved to be a very prolific source of natural products diversity, much of which is of microbial origin [64,65]. The study of the anthracimycin gene cluster [57] (Figure 4) is just one of many examples of bioactive natural products isolated from marine actinobacteria [66,67]. Problems with reproducing natural environmental conditions in the laboratory often make the cultivation and maintenance of marine isolates difficult, and so it is not surprising that investigators are increasingly adopting the approaches outlined here to capture and exploit the biosynthetic potential of marine micro-organisms [68,69,70,71,72,73]. A genus of marine actinobacteria particularly worth highlighting for yielding potentially pharmaceutically useful natural products is *Salinispora* [74]. *S. tropica* CNB-392 is the producer of salinosporamide A [75], a promising anticancer compound currently in clinical trials [66]. While some members of the *Salinispora* genus are amenable for culturing in the laboratory [76], heterologous expression of biosynthetic gene clusters from these species has also been achieved [77] in host strains of the model actinomycete *Streptomyces coelicolor* [78]. We predict that the application of now well-established techniques for genome mining will prove particularly effective for the analysis of biosynthetic gene clusters from these and other marine micro-organisms [1,7,8].

## 12. Concluding Remarks

One of the main misconceptions about NGS-derived genome assemblies is that they faithfully represent the complete sequence of a genome or even of a gene cluster. Wrongly assembled segments of a genome are quite frequent in draft assemblies obtained with SGS technologies like Illumina [79]. This poses a problem not only for specific research projects, but populates nucleotide sequence databases with poorly finished drafts of actinobacterial genomes, compromising automated computerised analyses, including homology searches and annotation. The problem of misassembly has been tackled using two main approaches: sequencing two different libraries of short and long insert size (e.g., the use of mate-pair reads in Illumina [80,81]) or using optical mapping technology to generate a genome-wide restriction map [82,83].

We should also be aware that a complete genome sequence may not be represented in a shotgun assembly, even if obtained with PacBio and in a single contig per replicon. The ends of linear replicons are particularly difficult to obtain without resorting to manual curation or even directed cloning and Sanger sequencing [84] although Illumina sequencing (and presumably other technologies using short insert-size libraries) does seem better able to approach the ends than PacBio [43]. It is also worth noting that the PacBio “corrected reads” may contain unassembled sequence which can, given their high accuracy, be readily identified by querying with a protein sequence (using the tBLASTn program).

454 pyrosequencing will be discontinued by Roche during 2016 [85], and the suitability of Ion Torrent for sequencing G+C rich genomes, has yet to be firmly established. Consequently, at the moment, Pacific Biosciences SMRT (PacBio) and Illumina MiSeq are the technologies of choice for *de novo* genome sequencing of actinobacteria. The final choice will depend on many factors, mainly the financing available and the goals of the sequencing project, but also the availability of each technology (Illumina is currently more widely available than PacBio, with bench-top instruments affordable by medium-sized laboratories and the presence of suppliers offering the technology). Based on our experience, PacBio currently provides a far better assembly of similar accuracy to Illumina MiSeq but at a higher cost; Illumina cannot currently match PacBio assembly using merely a short insert library and paired-end 2 × 300 nt reads.

The long reads provided by TGS PacBio, with a consensus accuracy of over 99%, makes it a very suitable technology capable of resolving the precise organisation of modular PKS and NRPS genes, even if we need to manually correct frame-shifts that are normally easily identified. As an alternative, a combination of Illumina paired-end (short insert size library) and mate-pair (long insert size library) has been reported to lead to correctly assembled modular PKS genes, but may rely on the use of customised assembly pipelines [81].

Based on our experiences, the aim of the project and the finances available, we recommend the following options for *de novo* actinobacterial genome sequencing:

1. If the aim is to obtain the highest quality genome sequence currently possible using NGS, we recommend a combination of PacBio and Illumina sequencing, with the unassembled Illumina data used to correct missing bases, and the assembled contigs used to add possibly missing sequences in the PacBio assembly.

2. If the aim is to obtain an accurate overall assembly of the genome or to obtain reliable single-contig assemblies of biosynthetic gene clusters that encode modular PKS or NRPS enzymes with a tolerable number of missing bases, then we recommend PacBio.

3. If the goal is to identify interesting putative biosynthetic gene clusters, and to design strategies for cloning or activating gene expression, or to identify a metabolic product by gene inactivation and comparative metabolite profiling, then Illumina MiSeq will be the most cost effective choice, with potentially just paired-end sequencing of a short-insert library sufficing.

With the increasing output of PacBio at lower cost, we do not favour the use of a limited amount of PacBio long read data to scaffold Illumina contigs or scaffolds, since this approach would not help with the likely misassembly problems in repetitive regions of the genome. Instead, the PacBio assembly should form the foundation for correction with Illumina reads or extension with Illumina contigs.

Using any of the mentioned technologies to obtain a reliable sequence, we can now make confident predictions of the likely products of PKS and NRPS gene clusters and more reliably apply strategies such as heterologous expression of synthetic gene clusters to identify the encoded metabolites [86]. A recent example of the crucial importance of obtaining an accurate sequence of a modular PKS gene cluster (obtained with PacBio) is the lobosamide biosynthetic gene cluster from the marine isolate *Micromonospora* sp. RL09-050-HVF-A. The gene cluster contains seven large highly repetitive modular PKS genes. Bioinformatic analysis of conserved residues in the amino acid sequences of the ketoreductase (KR) domains contained therein allowed the absolute configurations of the resultant hydroxyl groups to be accurately predicted and, together with complementary mass-spectrometry and NMR analyses, resulted in the determination of a precise molecular structure [87]. Marine actinobacteria are arguably one of the most promising, exciting and yet relatively untapped sources of novel natural products with the potential for development into a broad range of pharmaceutically-useful drugs [66]. We believe that the sequence-based approach that we have described here will play a major role in fulfilling this promise.

## Figures and Tables

**Table 1:** Summary of our group’s experience using NGS for genome mining. Only the *Streptomyces* *leeuwenhoekii* genome was published in full; for the others, only the relevant and confidently re-sequenced segments were published.

Microorganism	Technology	Year	Number of contigs^1^	N50 contig (nt)	Longest contig (nt)	Total sum of contigs	Total data	Lanes/ runs	Ref.
*Microbispora corallina* NRRL 30420	Solexa/ Illumina	Mid 2007	14395	163	4436	2.93 Mb	881 Mb	7	[37]
*Microbispora corallina* NRRL 30420	454	Mid 2008	7580 (3027)	1219	8913	4.64 Mb	28 Mb	1/4	[37]
*Streptomyces* sp. OH-4156	Solexa/ Illumina	Mid 2007	15,471	378	7830	8.5 Mb			[38]
*Planomonospora alba* NRRL 18924	454	Mid 2009	3066 (1618)	756	14,767	2.32 Mb	13 Mb	1/8	[40]
*Planomonospora alba* NRRL 18924	454	Mid 2011	1017 (944)	17,314	141,100	7.3 Mb	72 Mb	1/4	[40]
*Streptomyces chartreusis* NRRL 3882	454	2008	3112	4582	53,916	7.95 Mb	286 Mb	1/2	[41]
*Streptomyces bottropensis* DSM 40262	454	End 2010	463 (427)	40,440	183,403	8.85 Mb	115 Mb	1/4	[42]
*Streptomyces leeuwenhoekii* DSM 42122	Illumina MiSeq	Mid 2013	387 (279) (175 scaf.)^2^	59,284	157,225	8.1 Mb	712 Mb (1.25 Gb)	Full (500 cycles)	[43]
*Streptomyces leeuwenhoekii* DSM 42122	PacBio	End 2013	3	7,895,833	7,895,833	8 Mb	966 Mb	2 (3) cells	[43]

^1^ Total number of contigs is given first; when available, the number of contigs larger than 500 nt is given in brackets. ^2^ Number of scaffolds in which contigs were joined.

## Supplementary Materials

The following are available online at www.mdpi.com/link, Figure S1: Examples of insertions and deletions in PacBio and 454 assembled sequence in homopolymeric runs of G or C. Figure S2: Region posing difficulty for sequencing.

## Abbreviations

The following abbreviations are used in this manuscript:

NGS: Next Generation Sequencing
NRPS: Non-Ribosomal Peptide Synthetase
PKS: PolyKetide Synthase
PacBio: Pacific Biosciences SMRT technology
SGS: Second Generation Sequencing
TGS: Third Generation Sequencing
TIR: Terminal Inverted Repeat
