# Supplementary material for: Next Generation Sequencing of Actinobacteria for the Discovery of Novel Natural Products
Source: Mar Drugs. 2016 Apr 13;14(4):78. doi: 10.3390/md14040078 (PMC4849082; doi:10.3390/md14040078)
Supplement: Supplementary file 1 [file marinedrugs-14-00078-s001.pdf]

Supplementary Materials: Next Generation Sequencing of Actinobacteria for the Discovery of Novel Natural Products

Juan Pablo Gomez-Escribano , Silke Alt and Mervyn J. Bibb

A

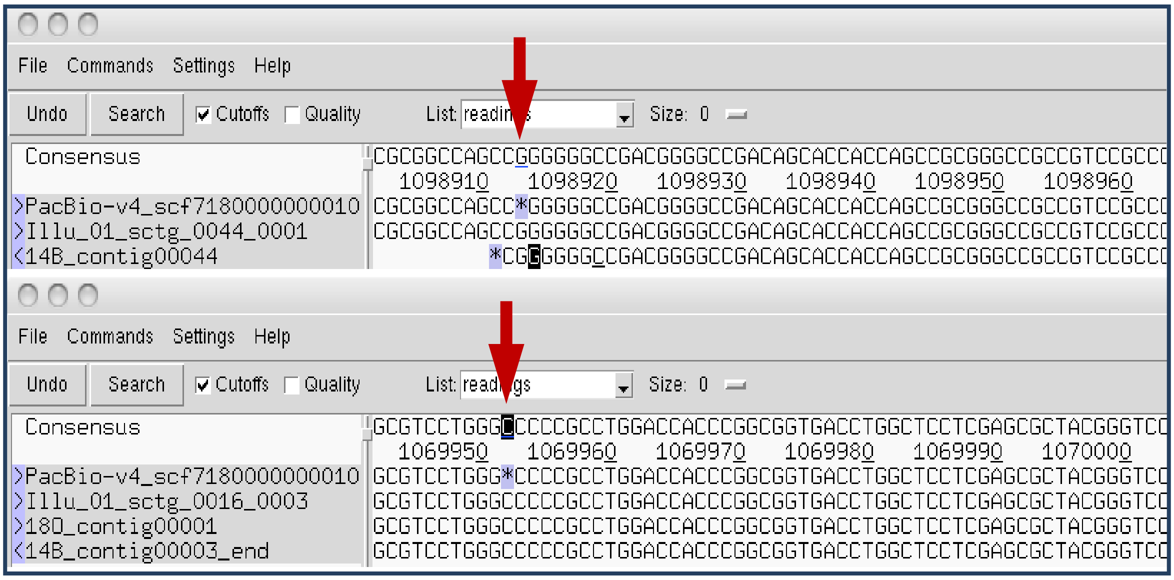

B

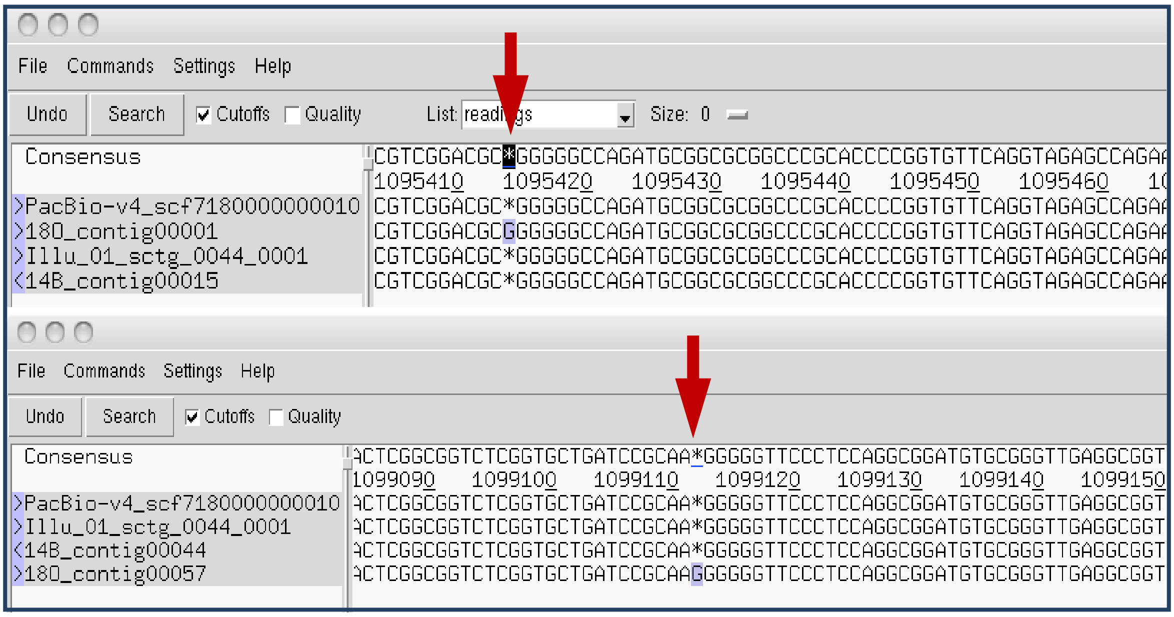

Figure S1. Examples of insertions and deletions in PacBio and 454 assembled sequence in homopolymeric runs of G or C. (**A**) Missing (deletion) of one G or C in PacBio. (**B**) Added (insertion) of one G at two positions of a 454 contig.

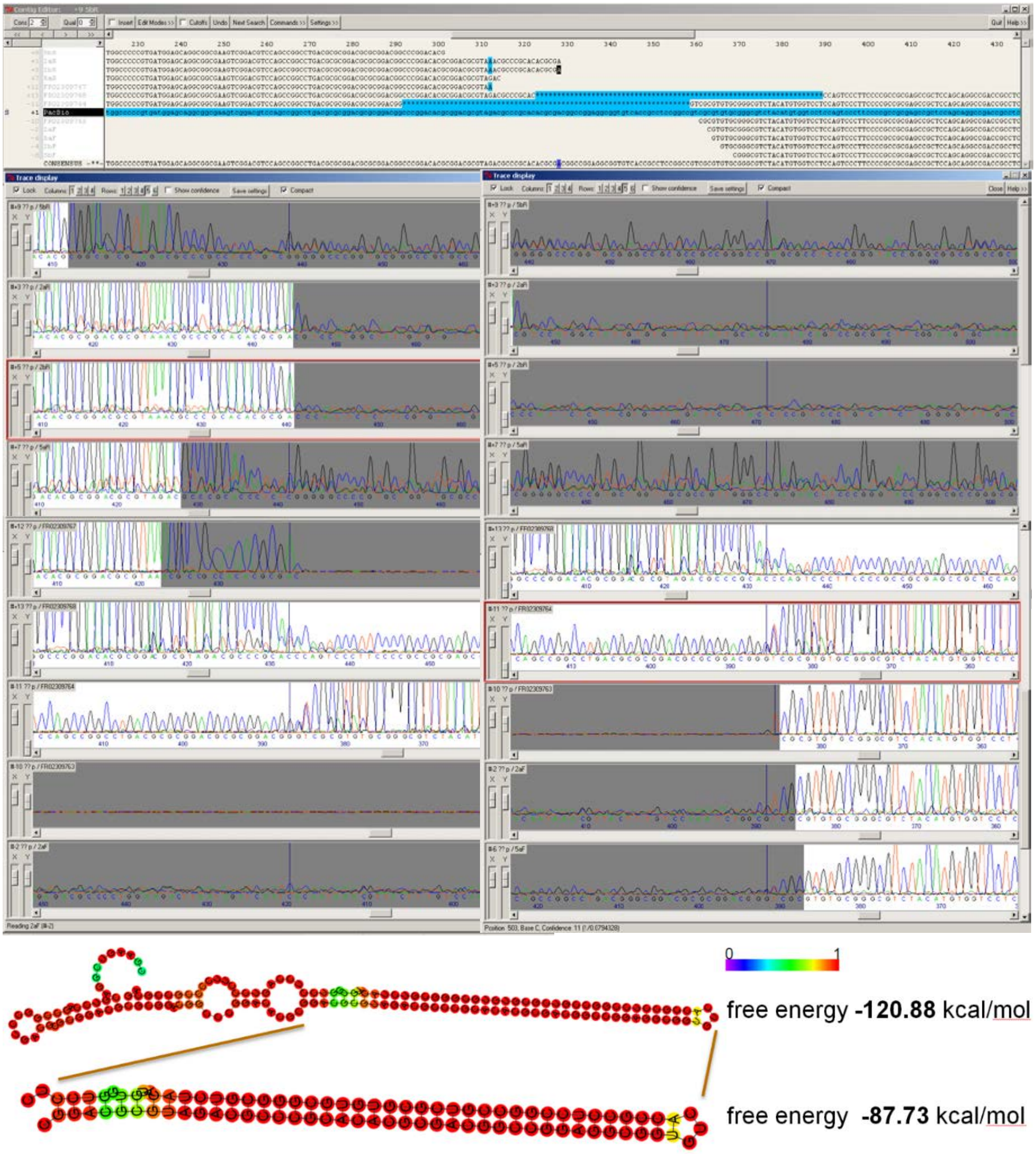

Structure and free energy calculated with RNAfold server (Gruber et al. Nucl. Acids Res. (2008) 36 (suppl 2): W70-W74.)

Figure S2. Region posing difficulty for sequencing. Region along which single stranded nucleic acid has the potential to form a strong secondary structure, and that neither Illumina nor Sanger sequencing could resolve, but PacBio did.
